# By executive order: The likely deadly consequences associated with a 90‐day pause in PEPFAR funding

**DOI:** 10.1002/jia2.26431

**Published:** 2025-02-25

**Authors:** Khai Hoan Tram, Jirair Ratevosian, Chris Beyrer

**Affiliations:** ^1^ Department of Medicine University of Washington Seattle Washington USA; ^2^ Duke Global Health Institute Duke University Durham North Carolina USA

1

On 20 January 2025, the first day of his second term in office, President Donald Trump issued an executive order instating a 90‐day pause on new U.S. foreign assistance, pending a review for alignment with U.S. foreign policy. Four days later, the U.S. State Department issued a “stop order” directive, expanding the pause to include a freeze on all foreign aid programmes, including the U.S. President's Emergency Plan for AIDS Relief (PEPFAR) [1]. By 1 February, PEPFAR received a limited waiver for life‐saving HIV care and treatment services and prevention programmes to prevent vertical transmission [2]. While this waiver signalled hope to millions, it did not release immediate funding to implementing partners, prolonging confusion and disruption on the ground [3, 4]. Ongoing uncertainty around PEPFAR funding has interfered with critical HIV programmes that rely on long‐term planning, making it impossible to operate effectively and sustain life‐saving services.

First announced under President George W. Bush in 2003 and reauthorized regularly since with bipartisan support in Congress, PEPFAR has been critical not only in the global response against the HIV epidemic but also in strengthening overall health systems in over 50 countries worldwide [5]. Over the past 21 years, PEPFAR has supported antiretroviral treatment (ART) for over 20 million people living with HIV (PLWH), including 566,000 children; reached 2.3 million adolescent girls and young women with comprehensive HIV prevention services; supported 6.6 million orphans, vulnerable children and caregivers; enrolled 2.5 million people on HIV pre‐exposure prophylaxis; provided 83.8 million people with HIV testing services; and directly supported 342,000 health workers [5]. Since its inception, PEPFAR is estimated to have saved 26 million lives and prevented 7.8 million infants from being born with HIV [5]. Additionally, in PEPFAR‐supported countries, new HIV infections have been reduced by half since 2010 [5].

The deadly consequences of even brief pauses in foreign aid cannot be overstated. At stake with the stoppage of U.S. foreign aid is PEPFAR's ability to continue its indispensable work of delivering life‐saving HIV treatment to millions of people and supporting local health system capacity. HIV treatment interruption leads to not only loss of virological control but also reversal of immune recovery for PLWH, the potential for viral resistance, the emergence of opportunistic infections, increased risk of tuberculosis and other co‐infections, and ultimately increased morbidity, mortality and onward transmission [6]. Based on previously described mathematical models of HIV epidemiology and intervention programmes in sub‐Saharan Africa, a 90‐day disruption of HIV treatment and care programmes modelled as discontinuation of ART to 50% of people is expected to lead to a median increase of 1.36 times the number of HIV‐related deaths over a 1‐year period [7]. Consequently, based on this model, we estimate that a 90‐day pause in funding and concomitant interruption of ART could result in over 100,000 excess HIV‐related deaths over the subsequent year. Disruption to HIV treatment for a greater percentage of people on treatment (e.g. 100% rather than 50%) or longer periods of treatment interruption (e.g. 6 months rather than 90 days) would certainly have even greater impacts.

In the above estimate, we have limited the calculation of excess HIV‐related deaths to only those resulting from interruption of treatment for 90 days, with the simplifying assumptions that a 90‐day pause in funds would equate to a 90‐day treatment interruption and that ART would be re‐initiated immediately at the end of the 90‐day period [7]. This estimate would be even higher if we considered the impact on HIV mortality that would result from the suspension of other PEPFAR programmes; for example, reduced HIV testing and diagnosis or a decrease in HIV prevention services or a decline in the prevention of vertical transmission. Indeed, the compounding impact that would result from the suspension of salaries for nearly 342,000 health workers across PEPFAR programmes in over 50 countries would further contribute to treatment disruptions. Additionally, we only attributed to PEPFAR a fraction of the excess HIV‐related deaths from disruption of services proportionate to the treatment coverage of PLWH supported by PEPFAR in each country (Table 1) [8, 9]. The actual number of PLWH affected by a 90‐day pause would most certainly be higher given the support that PEPFAR provides across health systems. Finally, though we report a projected increase in HIV‐related mortality, HIV incidence would also be expected to increase not only from disruption in HIV prevention programmes but also from the increase in transmission from greater population‐level viraemia. A recent modelling analysis from South Africa further supports these concerns, with 565,000 new infections projected over 10 years from a complete cutback in PEPFAR funding [10].

**Table 1:** Estimated excess HIV‐related deaths in PEPFAR‐supported countries from a 90‐day pause in PEPFAR programming

Country	PEPFAR‐supported PLWH currently on ART (FY 2024)^a^	PLWH receiving ART (2023)^b^	% supported by PEPFAR (max 100%)^c^	HIV‐related deaths (2023)^d^	Estimated excess HIV‐related deaths from a 90‐day pause in PEPFAR funding^e^
Angola	27,000	160,392	0.17	12,000	727
Botswana	195,000	339,716	0.57	3900	806
Burundi	70,000	74,096	0.94	1200	408
Cameroon^*^	415,000	413,135	1.00	7400	2664
Cote d'Ivoire	266,000	305,545	0.87	9500	2977
DRC	185,000	444,592	0.42	11,000	1648
Eswatini	208,000	213,416	0.97	3100	1088
Ethiopia	521,000	513,990	1.00	10,000	3600
Kenya	1,339,000	1,336,681	1.00	21,000	7560
Lesotho	245,000	241,462	1.00	4000	1440
Malawi	944,000	896,805	1.00	11,000	3960
Mozambique	1,976,000	2,088,982	0.95	44,000	14,983
Namibia	217,000	202,605	1.00	3700	1332
Nigeria	1,575,000	1,735,808	0.91	45,000	14,699
Rwanda	134,000	221,149	0.61	2600	567
South Africa	5,334,000	5,936,501	0.90	50,000	16,173
South Sudan	64,000	67,455	0.95	5600	1913
Tanzania	1,539,000	1,389,883	1.00	25,000	9000
Uganda	1,439,000	1,244,193	1.00	20,000	7200
West Africa Region 1^#^	132,000	197,554	0.67	7300	1756
West Africa Region 2^##^	99,000	311,659	0.32	19,200	2196
Zambia	1,295,000	1,273,804	1.00	17,000	6120
Zimbabwe	1,215,000	1,233,934	0.98	19,000	6735
TOTAL					**109,552**

^a^ People living with HIV (PLWH) currently on antiretroviral therapy supported by PEPFAR (FY2024, Source: amfAR).

^b^ Total PLWH currently on ART in 2023 (Source: UNAIDS).

^c^ Estimated % of PLWH on ART that is supported by PEPFAR by country, max 100% (Source: authors’ calculation).

^d^ Reported HIV‐related deaths in 2023 (Source: UNAIDS).

^e^ Estimated excess HIV‐related deaths from 90‐day interruption in ART to 50% of the population (Source: authors’ calculation).

* Cameroon's reported total PLWH on ART and HIV‐related deaths are from 2022 (Source: UNAIDS).

^#^ West Africa Region 1 (PEPFAR) includes Benin, Burkini Faso, Senegal and Togo.

^##^ West Africa Region 2 (PEPFAR) includes Ghana, Liberia, Mali, Sierra Leone, Zambia and Zimbabwe.

PEPFAR is the cornerstone of U.S. global health efforts and an outstanding example of the generosity and compassion of the American people. PEPFAR's impact reaches far beyond its humanitarian mission, creating a multifaceted web of benefits that bolster U.S. economic interests, strengthen diplomatic influence and enhance global health security [11, 12, 13].

The current limited waiver for PEPFAR has done little to mitigate the widespread impact of the aid pause, which has caused clinic closures and disrupted life‐saving services, jeopardizing global progress in HIV prevention and treatment [14]. Further, most HIV prevention efforts under PEPFAR remain unauthorized, leaving key programmes suspended and raising the prospect of increasing HIV acquisition and emerging drug resistance [2]. With PEPFAR's authorization set to expire on 25 March 2025, and the unknown results and consequences of the foreign aid review, stakeholders face an urgent need to mobilize and advocate for the programme's future sustainability [15].

By our conservative estimate, the human cost of the new administration's proposed 90‐day policy review would be at least 100,000 lives lost over 1 year. We call for an immediate re‐instatement of all funding for PEPFAR to continue to provide essential services and renewed U.S. leadership in global health in alignment with U.S. interests and values.

## AUTHORS’ CONTRIBUTIONS

KHT conceptualized the work, led analysis of the data and wrote the first draft of the manuscript. JR and CB provided critical feedback and contributed to writing and editing. All authors read and approved the final manuscript.
